# Effect of Grouping, Segmentation, and Vestibular Stimulation on the Autokinetic Effect

**DOI:** 10.1177/2041669517752716

**Published:** 2018-01-17

**Authors:** Vilayanur S. Ramachandran, Chaipat Chunharas, Rachel Croft, Nader Batal

**Affiliations:** 8784 University of California San Diego, La Jolla, CA, USA

**Keywords:** autokinetic effect, eye movements, grouping, gestalt grouping, size constancy

## Abstract

We report some new observations on what could be regarded as the world’s simplest visual illusion—the autokinetic effect. When a single dim spot of light is viewed in a completely dark room, it moves vividly in random directions. During steady fixation, perhaps subtle eye movements cause the image to move and a failure to correct for this using eye movement command signals leads to motion perception. This is especially true because eye muscle fatigue can lead to miscalibration. However, if two dots are shown, they often move independently in different directions, which negate the eye movement theory. In addition, two lines defining a single cross sometimes uncouple and slide past each other and the fragments composing a hidden object move independently until they click in place and the whole object is perceived—implying that the illusion occurs relatively late in visual processing. Finally, the effect is modulated by vestibular stimulation; anchoring your sense of self may be a prerequisite for binding features into coherent objects.

Stimuli were small strips of luminous paper mounted on a white wall such that in complete darkness only the spot(s) were visible - allowing us to observe the autokinetic effect (Gregory and Zangwill, 1963; Schweizer 1857). Subjects viewing the display reported seeing movement—either immediately or after a latency. As with many perceptual illusions, a small proportion of people never (or very rarely) experience the effect (6 out of 34 total participants).

Next, subjects were asked to watch two adjacent dots (with one eye closed) and to indicate on the keyboard whether the dots moved in unison (holding one key) or were uncoupled (holding another key). We report the results of two formal experiments along these lines followed by four informal demonstrations.

## Experiment 1: Effect of Horizontal Separation Between Dots

**Figure 1.**
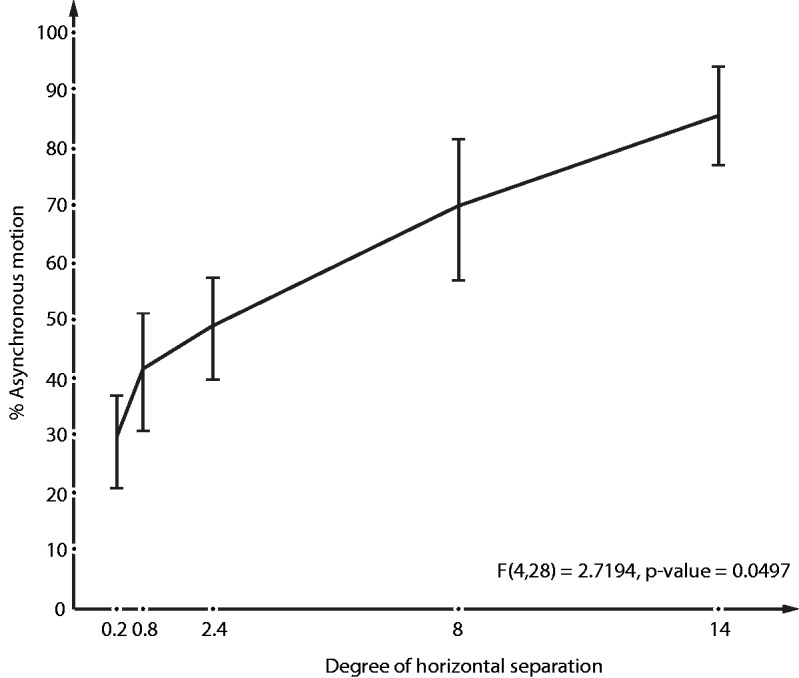
The percentage of uncoupled motion increased as the two dots were placed farther apart, suggesting the illusion occurs after grouping by proximity.

We presented two adjacent dots each subtending 0.35 degrees with separation of 0.25, 1, 3, 10, and 17.5 degrees. Nine naïve subjects were asked to view the display with one eye and press the buttons on a keyboard to indicate whether the dots moved in unison or were uncoupled (we could infer by default, the proportion of time they appeared stationary). We found the dots were uncoupled and progressively more often as the separation between dots increased—casting further doubt on any simple version of the theory that the illusion is based on eye movements or reafference from commands (because the single eyeball cannot move simultaneously in multiple directions) (Figure 1).

## Experiment 2: Size Constancy

**Figure 2.**
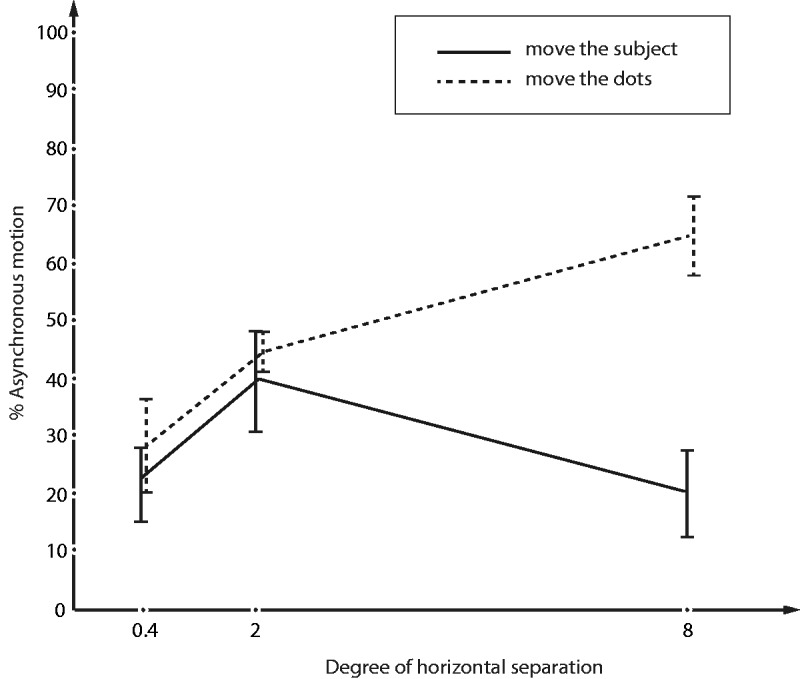
As in Experiment 1 (Figure 1), the dots were seen to uncouple more when they were far apart from each other (dotted line). But if the subjects moved closer to the dot pairs they did not uncouple equivalently—showing a partial size constancy correction (solid line).

The perceived distance between two horizontally separated dots remains relatively constant as you walk toward them; despite the rapidly increasing size on the retina. This is because the brain takes into account distance cues and applies a correction to calculate the real physical separation. We wondered, therefore, whether this effect occurs prior to, or after, the autokinetic effect. In Experiment 1, we showed a monotonic relationship between horizontal separation and duration of uncoupling. Comparing the results of the present Experiment 2 (new nine naïve subjects) to Experiment 1, it seems clear that moving yourself closer to the display does not lead to equivalent uncoupling because the brain uses subtle depth cues (e.g., from small amount of stray light as well as nonretinal cues such as accomodation and overall cognitive inference about the room layout) to infer that the horizontal separation has not actually changed. The net result is the degree of synchrony/asynchrony is roughly half way between perfect constancy and no constancy—compelling evidence proving that effect does not occur early in sensory processing (Figure 2).

## Demonstration 1: Grouping Effects

**Figure 3.**
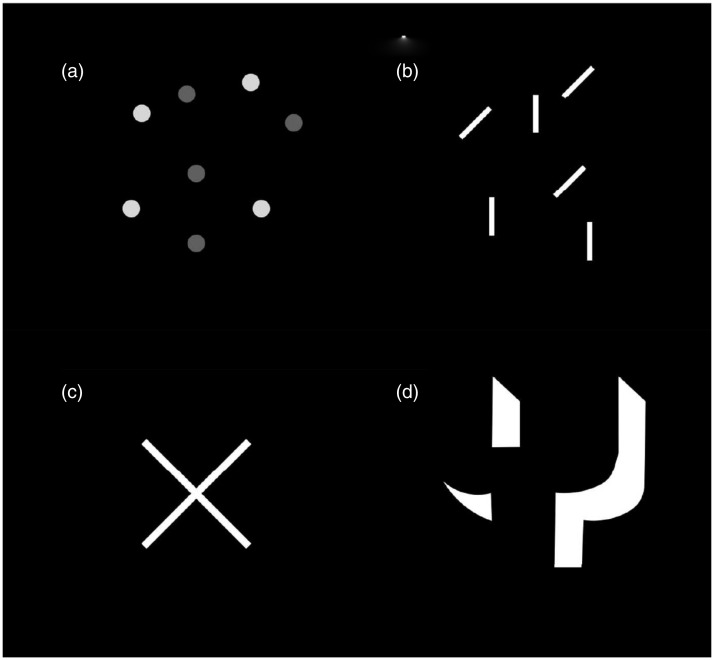
Grouping by contrast (a) and grouping by line orientations (b). (c) Two perpendicular lines moved independently on many trials. (d) The fragments moved independently until they saw the 3D block letter “Y.”

We have seen in Experiments 1 and 2 that grouping by proximity results in the dots moving synchronously whereas if the dots are far apart they uncouple and wander in different directions. We wondered if other types of grouping also play a role. Figure 3(a) depicts grouping by contrast and Figure 3(b) by orientation of lines—in both cases, grouping by similarity resulted in uncoupling and independent movement of the two clusters. Four naive subjects experienced this.

## Demonstration 2

If you look at (say) two orthogonal lines forming an X shape in a fully lit room, you see it exactly as it is. But when you view two luminous lines in darkness—they uncouple and almost slide off each other! This implies that illusory motion signals are differentially applied to the lines prior to the solution to the aperture problem. In addition, if one voluntarily stratified the lines mentally—then the nearer of the two lines always appeared to slide over the farther (Figure 3(c)). Thus, image segmentation (aperture problem) and top-down processing can strongly modulate autokinetic effect.

## Demonstration 3

The top-down component was especially striking in the experiment using an illusory three-dimensional (3D) Y shape defined by attached shadows (Figure 3(d)). The figure is initially seen as mere irregular fragments by most people but eventually a 3D “Y” is seen. Intriguingly, if luminous fragments are used, subjects initially see them moving incoherently; though not always. But as soon as the fragments “click” into place forming a 3D “Y”; they always move together as a single object.

## Demonstration 4

One might expect that binding of multiple attributes of the visual scene into a stable coherent percept would also require prior anchoring of the observing self in one’s body scheme—which in turn is strongly modulated by vestibular input. Indeed, the strategic location of vestibular cortex in the vicinity of polymodal parietal cortex makes it an ideal candidate for both anchoring and binding; for example, patients with parietal lesions often have somatoparaphrenia (denial of arm; Ramachandran, 2003) as well as illusory feature conjunctions (caused by a failure of binding; Robertson, Treisman, Friedman-Hill, & Grabowecky, 1997). Although vestibular modulation of autokinetic effect has been observed before (Gregory & Zangwill, 1963; Nelson, 2001), we were curious whether the degree of movement, coupling, and feature binding would be influenced by the procedure.

We used a portable noninvasive galvanic vestibular stimulation to modulate vestibular function in six naive subjects. Five subjects reported that there was substantially more illusory motion—and of these, four reported more coherence and binding during the stimulation; most commonly a rocking or seesaw motion at 1 Hz (curiously, one subject reported less binding). These informal observations suggest that vestibular modulation does indeed occur. However, additional experiments are needed to confirm this.

We suggest that the brain creates multiple drafts of parallel sensory inputs on temporary sketch pads before integrating them at various stages to create a subjective impression of coherence and stability. In a room full of objects, the noise from parallel sources gets averaged out to increase signal-to-noise ratio. However, in a cue impoverished environment with a single luminous dot, such noise cancellation fails and the dot is free to wander.

Taken collectively, these experiments on autokinetic effect allows us to explore the laws of cue integration and provide a novel approach to the binding problem and its relevance to consciousness. In addition, far from being a curiosity, the illusion has relevance to our war and defense; during the Gulf War, American scouts perceived a whole battalion of enemy tanks move toward them and launched a counterstroke, failing to realize it was autokinetic effect. Had the realization come any later, we might now be facing nuclear winter and would not have written this article.
